# The Bonding Force of Juvenile Idiopathic Arthritis-Families Transforming through JIA: A Qualitative Research Study

**DOI:** 10.31138/mjr.080424.qsr

**Published:** 2025-05-22

**Authors:** Foteini Galani, Maria Magdalini Delliou, Christina Papachristou

**Affiliations:** 1 Aristotle University of Thessaloniki, Thessaloniki, Greece; 2 Parents’ and Caregivers’ Association of Children with Chronic Rheumatic Diseases, Thessaloniki, Greece

**Keywords:** juvenile idiopathic arthritis, qualitative study, family relationships, family everyday life, coping mechanisms

## Abstract

**Objectives:**

Central aim of this research was to investigate the experience of parents with a child diagnosed with Juvenile Idiopathic Arthritis (JIA) and the interplay of JIA, family dynamics, everyday life, and the developing coping mechanisms to deal with potential life changes.

**Methods:**

A qualitative psychology-based research methodology was applied. Data were collected through online in-depth semi-structured interviews with parents of one offspring with JIA. The anonymised interview transcripts were analysed following the principles of interpretative phenomenological analysis. Nine parents (M:F, 3:6) aged 39–51 years (mean=47), were included in the study with their children having the diagnosis of different JIA subtypes.

**Results:**

JIA appears to have led our sample to the creation of strong bonding between parents, siblings, and the patient (9/9). Additionally, JIA altered the family members’ daily life by shifting their focus on the disease management (9/9). The upcoming stress led parents to develop a variety of coping mechanisms (9/9) with 3/9 parents sought reliable information from health professionals, 7/9 looked for psychological support and 6/9 shared their experience with other JIA affected families. Impressively, 5/9 reported that JIA eventually has a positive impact on their family, with JIA minors presented as disciplined and empathetic fighters (4/9).

**Conclusion:**

JIA was found to be a great challenge from the parents’ perspective, especially during the post-diagnosis period. However, most of the interviewed family members gradually accommodated to JIA and acknowledged even its beneficial contribution.

## INTRODUCTION

JIA is a chronic medical condition, being the predominant type among juvenile rheumatic diseases.^1^ It is an umbrella term including six subtypes which are differentiated based on their symptoms’ duration and severity, their diagnostic process and thus their line treatment.^2^

The chronic element of this medical condition and its impact to the everyday life of patients, shift the research focus also to the investigation of their life quality in terms of daily pain, anxiety and psychosocial challenges.^3^ Furthermore, childhood chronic diseases seem to have additional impact on the family environment, on a psychological, social, and financial level. Hence, an investigation of both patients’ and family’s life quality appears of high importance.^4^

Despite the high volume of findings regarding the impact of JIA on the affected patients, Greek data from a family perspective are currently limited. Thus, the focus of this study is to investigate the experience of families with a child diagnosed with JIA, as well as the interplay between JIA, family dynamics, and everyday life, and to reveal the coping mechanisms developed by their parents to deal with the potential life changes. Its significance is based on understanding JIA’s influence on the rest of family members in order to increase the awareness among healthcare providers and thus implement the appropriate interventions tailored to their needs.

## METHODS

Strictly adherent to ethical guidelines, a qualitative research methodology and specifically the interpretative phenomenological analysis was chosen to investigate participants’ experience and to address research questions.^5^ Data collection was based on semi-structured online interviews with parents of child diagnosed with JIA, which were conducted via Zoom platform. Semi-structured interviews were chosen to best capture participants’ experience with the open-ended nature of the questions allowing for greater expression as they are less directive.^6^ The focus is on the personal perspective of each participant, which varies by context and speaker.^7^ The interview guide of this study was constructed especially for the purpose of it. Its domains were based on extensive literature review, with emphasis on the aspects that seem to be generally affected by chronic diseases. The quality and language of the interview guide was initially evaluated by the study’s supervisor and validated during the first interview which was treated as a pilot one. The domains of the interview guide were: a. the experience of JIA diagnosis throughout the time, b. the relationship between parents, JIA-diagnosed minors as well as their siblings, c. the impact of JIA on their everyday life, and d. the coping mechanisms applied to deal with the onset of JIA. Interviews were conducted in Greek language and each one lasted 25 to 60 minutes with a median of 40 minutes. Interview audio files were transcribed and then analysed based on the principles of interpretive phenomenological analysis.

Following a purposive sampling method, participants had to be parents of a minor with JIA, who had JIA diagnosis for at least two years. However, only one of the two parents could take part in the study, and JIA had to be the only chronic condition among family members. To ensure participants’ diversity, sample was deliberately differentiated in terms of sibling’s number and in terms of the minors age when diagnosis (i.e. one to four, five to ten, and eleven to sixteen years old).

The final sample consisted of nine parents of children with a JIA’s sub-type, with six of them being females and three males, and an average age of forty-seven years. With regards to their educational level, 3/9 had a high-school diploma, 5/9 a bachelor’s degree and 1/9 a master’s degree. As per the gender of JIA children, seven were girls and two were boys, with an average age of thirteen years. All JIA subtypes were reflected in the sample except for the undifferentiated one. Although the functionality of JIA children was not set as an inclusion criterion, the diversity in terms of JIA sub-types resulted in a balanced sample. Due to the requirement for the children to be diagnosed with JIA at least two years prior to the study most of them showed a moderate to high functionality level. However, this was poorer in more complex cases where the number of joints involved was greater. Data were analysed by the authors following the interpretive phenomenological analysis method which resulted in six main domains revealed, as shown in **Appendix Table 1**.

## FINDINGS

Six main themes were revealed through the analysis of the interview transcripts that describe the experience of families with a minor diagnosed with JIA (**Appendix Table 1**).

### Theme 1: Changes in family dynamics

JIA led to the creation of stronger bonds between parents, siblings, and the affected patient (9/9) which helped families when dealing with JIA’s concerns, while also resulted to the effective management of the disease by the affected children. The non-affected siblings are described to have developed their ability for empathy, while parents reported an increased support in their everyday tasks by their extended family members. This combination boosted the resilience of family and helped them collectively counterbalance JIA’s difficulties.

At the same time, JIA created a feeling of confusion within families, especially in the post-diagnosis period and frequently raised disagreements on medication-related issues (4/9). The feeling of confusion was reported by parents who expressed at the announcement of the diagnosis that they were unaware of JIA, believing arthritis can only be present in adulthood. Most of the parents were also confused because they initially attributed JIA symptoms to a temporary condition, only to later realise its chronic implications.

JIA shifted parental focus to the affected minor as a protective strategy, which generated neglect and jealousy emotions between parents and rest of family members.

### Theme 2: Changes in everyday life

Parents reported increased responsibilities which resulted from their child’s medical condition. The most common change being referred was their shift of focus to doctors’ appointments and medication needs as well as to the special nutrition their child had to undertake. Half of them reported as a major daily change the obligation for the affected minors to modify their activities based on the needs and obstacles of this chronic disease in their effort to avoid potential severe injuries, which might worsen their condition. As an example, one parent mentioned the avoidance of sport activities such as skiing and football, while others paused their children from kindergarten in order to prevent them from virus infection during immunosuppression periods.

### Theme 3: Difficulties

Parents struggled in finding the appropriate healthcare specialists and doctors in terms of experience, physician approach and personality. Frequent obstacle was shortages in medication along with frequent pharmaceutical scheme changes which were needed until the most effective one to get found. In addition, parents had to also deal with children’s resistance to both medical examinations and to the specific diet they had to follow which most of the times put them against to each other.

### Theme 4: Emotional reactions

Especially throughout the post-diagnosis period, parents suffered from anxiety emotions, due to symptoms’ permanence, complexity and their unpredicted progress. They were highly concerned regarding the affected minors’ vulnerability which is maximised throughout disease’s outbreak periods. In the meantime, one common stress factor among the participants was the severity of medication that their children had to take to manage JIA’s symptoms and its potential side-effects. From a psychological perspective, 5/9 parents reported blaming feelings to themselves as they have potentially inherited JIA’s idiopathic element to their child. However, the initial deadlock was replaced by relief emotions which resulted from a suitable medication and healthcare support.

### Theme 5: Coping mechanisms

To deal with the upcoming stress, parents sought reliable information from healthcare professionals to obtain detailed insights regarding the condition and its effective management. This, in combination with individual psychotherapy sessions, minimised the fear caused by information online searching. Their anxiety emotions relieved by sharing their experience with other JIA affected families through participating or even initiating parents’ associations. In these associations parents exchanged advice and insights, which also allowed them to forecast potential difficulties and find the appropriate solution in advance (6/9). Lastly, another approach which was effective when dealing with JIA was that parents maintained a positive attitude towards minor daily issues and developed easily understood ways to present JIA impact to their affected and non-affected minors via intrafamilial discussions.

### Theme 6: JIA’s positive impact

5/9 participants reported that JIA eventually had a positive impact on their family by teaching them to re-evaluate and re-prioritise problems and to appreciate small moments of happiness. They described JIA minors as fighters and role models who developed resilience and discipline due to their disease responsibilities and restrictions, while showing increased empathy levels by understanding and accepting each person’s diversity.

## DISCUSSION

JIA was found to influence the family life and its daily routine and thus it requires an appropriate management within the family context, which has been initially explained by Goldstein and Kenet from a homeostatic perspective.^8^ Although the strengthened connection among family members due to a chronic illness has been also described by Chesler et al. and Waite-Jones and Madill as a conscious effort by the non-affected family members, participants in this study presented it as a natural outcome.^9,10^ The affected minors were presented to need special attention while their medical issues were central in family’s everyday life. This led the rest of family members to invest on understanding their medical and non-medical needs and on helping them to reduce the disease caused fatigue.

The adherence to medical instructions and the resistance of JIA minors to the injectable form of medication frequently raised disagreement between them and their parents who were strongly affected emotionally. Parents explained their emotional conflict, as most of the times they felt to hurt their children through the injectable treatment form. Thus, they were afraid of this being misunderstood by their children as a sign of lack in loving them. Additionally, the parental adherence to JIA minors, forced their siblings to express neglect emotions and feign JIA symptoms to gain their attention.

Regardless of changes’ magnitude, 9/9 participants adopted a combination of management strategies which confirm previous findings in terms of seeking reliable information from health experts to reduce the initial anxiety caused by disease unawareness and online searching of information, and of participating or initiating parents’ associations.^11,12^ A new finding of this study is the disease management by initiating an intrafamilial discussion, and by consciously adopting a positive perspective towards the disease and daily issues in a parental effort to act as role models to their affected and non-affected children.

Despite the initial discomfort due to the changes and difficulties caused by JIA within the affected family, parents found to adapt to this new condition, and once accepting its permanent element they begin to effectively manage it. As a first noticed finding within the current existing literature, participants in this study revealed the disease’s positive impact in terms of re-evaluating their daily difficulties and appreciating moments of happiness. Previous findings placed chronically ill patients in a passive position, and described them as isolated with an increased psychological vulnerability to depression and post-traumatic stress disorder.^13,14,15^ In contrast to those findings, parents who participated in this study described children with JIA as disciplined fighters and role models for the rest of family members who developed empathy and acceptance towards others’ different and unique characteristics.

## LIMITATIONS

In terms of the methodology, one limitation of this study can be based on its small sample size, a number which potentially restricts findings’ generalisation while the interview-based approach could potentially lead to obtain socially desirable answers. Also, the acceptance finding of JIA by 9/9 participants in this study may result from the long period since their minors’ diagnosis, which was set as an eligibility criterion for their participation. Furthermore, another results bias might be that families that were significantly affected by JIA and have not managed to cope with its challenges have probably avoided participating in the study.

## CONTRIBUTION

This study fills the literature gaps by exploring JIA’s experience and impact within Greek families along with their management strategies. It also reveals the positive aspect of chronic diseases because of the valuable lessons they teach to the affected and non-affected family members such as the focus on the important things in life. The final stage of acceptance is expected to help parents whose children just received the diagnosis by relieving the initial stress and by forecasting a positive outcome in terms of JIA’s effective integration into their daily life. By revealing the hidden and unhidden needs of parents with JIA diagnosed children, this study will contribute to their effective management from heath specialists as they can provide the appropriate support from an earlier stage. Based on participants’ need to share their personal experience with other JIA parents, the creation of a greater number of parents’ associations will be beneficial for them in order to cope with JIA emotional and daily challenges. However, future research is vital to focus on families with recently diagnosed JIA minors in order to validate the implication of those findings among this audience.

## CONFLICT OF INTEREST

The authors declare no conflict of interest.

## FUNDING

The authors declare that no funding was received for this research.

## RELATED CONGRESS

Research outcomes were also presented in the 29^th^ European Paediatric Rheumatology Congress which took place in Rotterdam from 28^th^ September until 1st October 2023.^16^

## ETHICS APPROVAL

This research was conducted in accordance with Article 8 requirements as outlined by the Ethical Committee of the Department of Psychology at Aristotle University of Thessaloniki.

## APPENDIX

### Appendix

**Table 1:**

**Themes**	**Illustrating quotes**
Theme 1: Changes in family dynamics	“JIA was a bonding opportunity for us. Since diagnosis, we support each other more often and we deal every minor issue together.” “Since my daughter has been JIA diagnosed, she had my whole affection and although I didn’t mean to neglect my husband and her siblings, it frequently raised jealousy emotions from their side and arguments between us. But in the end, they all seemed to understand her needs and most of the times they helped her to overcome her daily difficulties.”
Theme 2: Changes in everyday life	“Since my son diagnosed with JIA, I had to modify my everyday schedule according to his, especially when it comes with his medication…Something that stressed me a lot was related to sport activities. I wasn’t meant to stop him by following his dream, but I tried to show him another path, safer for his health because I was afraid of a potential relapse.”
Theme 3: Difficulties	“In the beginning I was suffering of finding the right doctor and we had to change lots of them to find a team that is tailor made to our needs. Because for us, it was of the same importance their experience with JIA alongside with their approach to our child… Another major issue was the medication. Not being able to find the appropriate medication for your child because of that being in shortage, makes your everyday life even harder. But there were also cases when we had that treatment, but our daughter was crying in panic to avoid the injection.”
Theme 4: Emotional reactions	“In the beginning, I was shocked and confused because I wasn’t aware of that disease. And as JIA is a chronic condition, I’m still afraid of future relapse periods such as during pregnancy.” “In our case, JIA was the solution to our daily problems. Since there was a chance for my daughter to suffer from other much more serious conditions, the announcement of JIA diagnosis was a relief for us. Now that we know the reason behind these symptoms, we can effectively manage them.”
Theme 5: Coping mechanisms	“My first thought was to search for this disease on the internet. However, this ended up increasing my anxiety and fear levels, which were finally reduced by our experienced and trustworthy rheumatologist in combination with the mental support we received in terms of psychotherapy.” “The most helpful for me was to be in frequent contact with other parents of JIA affected minors to share our tips and advice. Especially them whose child was diagnosed a couple of years ago was my chance to visualise mine in the future. However, it was also vital to explain the disease within the family in an understandable way for my children such as by referring to the injection part as a body thermometer.”
Theme 6: JIA’s positive impact	“At the end, JIA converted my child into a great fighter who put higher effort compared to his peers to achieve his goals. And because he is always aware of the possibility that JIA can also have some bodily visible symptoms, he couldn’t ever make fun of any other person. And when you see your children fighting with these daily issues, the only thing you can do as a healthy adult is to appreciate even a small moment of happiness.”
